# Neuroimaging and Pathology Biomarkers in Parkinson’s Disease and Parkinsonism

**DOI:** 10.3390/brainsci16010110

**Published:** 2026-01-19

**Authors:** Roberto Cilia, Dario Arnaldi, Bénédicte Ballanger, Roberto Ceravolo, Rosa De Micco, Angelo Del Sole, Roberto Eleopra, Hironobu Endo, Alfonso Fasano, Merle C. Hoenig, Jacob Horsager, Stéphane Lehéricy, Valentina Leta, Fabio Moda, Maria Nolano, Tiago F. Outeiro, Laura Parkkinen, Nicola Pavese, Andrea Quattrone, Nicola J. Ray, Martin M. Reich, Irena Rektorová, Antonio P. Strafella, Fabrizio Tagliavini, Alessandro Tessitore, Thilo van Eimeren

**Affiliations:** 1 Fondazione IRCCS Istituto Neurologico Carlo Besta, Department of Clinical Neurosciences, Parkinson and Movement Disorders Unit, 20133 Milano, Italy; roberto.eleopra@istituto-besta.it (R.E.); valentina.leta@istituto-besta.it (V.L.); 2 Clinical Neurophysiology, IRCCS Ospedale Policlinico San Martino, 16132 Genova, Italy; dario.arnaldi@gmail.com; 3 Department of Neuroscience (DINOGMI), University of Genova, 16132 Genova, Italy; 4 Université Claude Bernard Lyon 1, CNRS, INSERM, Centre de Recherche en Neurosciences de Lyon, CRNL U1028 UMR5292, PATHPARK, F-69500 Bron, France; benedicte.ballanger@cnrs.fr; 5 Center for Neurodegenerative Diseases-Parkinson’s Disease and Movement Disorders, Unit of Neurology, Department of Clinical and Experimental Medicine, University of Pisa, 56126 Pisa, Italy; roberto.ceravolo@unipi.it; 6 Neurology Unit, Department of Clinical and Experimental Medicine, University of Pisa, 56126 Pisa, Italy; 7 Department of Advanced Medical and Surgical Sciences, University of Campania “Luigi Vanvitelli”, 80138 Naples, Italy; rosita.demicco@gmail.com (R.D.M.); alessandro.tessitore@unicampania.it (A.T.); 8 Department of Health Sciences, University of Milan, 20122 Milan, Italy; angelo.delsole@unimi.it; 9 Centro Diagnostico Italiano (CDI), Via Simone Saint Bon 20, 20147 Milan, Italy; 10 Advanced Neuroimaging Center, Institute for Quantum Medical Science, National Institutes for Quantum Science and Technology, Chiba 263-8555, Japan; endo.hironobu@qst.go.jp; 11 Edmond J. Safra Program in Parkinson’s Disease, Morton and Gloria Shulman Movement Disorders Clinic, Toronto Western Hospital, UHN, Toronto, ON M5T 2S8, Canada; alfonso.fasano@uhn.ca; 12 Department of Biomedical Sciences, Humanitas University, Via Rita Levi Montalcini 4, 20090 Milan, Italy; 13 IRCCS Humanitas Research Hospital, via Manzoni 56, 20089 Milan, Italy; 14 Multimodal Neuroimaging, Department of Nuclear Medicine, University Hospital and Medical Faculty, University of Cologne, 50937 Cologne, Germany; merle.hoenig1@uk-koeln.de (M.C.H.); thilo.van-eimeren@uk-koeln.de (T.v.E.); 15 Molecular Organization of the Brain (INM-2), Institute of Neuroscience and Medicine, Research Center Jülich, 52428 Jülich, Germany; 16 Department of Nuclear Medicine, Aarhus University Hospital, 8000 Aarhus, Denmark; jacobnls@rm.dk (J.H.); nicola.pavese@newcastle.ac.uk (N.P.); 17 Lundbeck Foundation Parkinson’s Disease Research Center (PACE), Aarhus University, 8000 Aarhus, Denmark; 18 Paris Brain Institute-ICM, Center for NeuroImaging Research (CENIR), Sorbonne University, Inserm, CNRS, 75013 Paris, France; stephane.lehericy@gmail.com; 19 Department of Neuroradiology, Pitié-Salpêtrière Hospital, AP-HP, 75013 Paris, France; 20 Department of Basic and Clinical Neuroscience, King’s College London, Institute of Psychiatry, Psychology and Neuroscience, The Maurice Wohl Clinical Neuroscience Institute, London SE5 8AF, UK; 21 Department of Medical Biotechnology and Translational Medicine, Università degli Studi Milano, 20122 Milan, Italy; fabio.moda@istituto-besta.it; 22 Unit of Laboratory Medicine, Laboratory of Clinical Pathology, Fondazione IRCCS Istituto Neurologico Carlo Besta, 20133 Milan, Italy; 23 Department of Neuroscience, Reproductive Sciences and Odontostomatology, University “Federico II” of Naples, 80131 Naples, Italy; maria.nolano@icsmaugeri.it; 24 Skin Biopsy Laboratory, Istituti Clinici Scientifici Maugeri, IRCCS, 82037 Telese Terme, Italy; 25 University Medical Center Göttingen, Department of Experimental Neurodegeneration, Center for Biostructural Imaging of Neurodegeneration, 37075 Göttingen, Germany; tiago.outeiro@med.uni-goettingen.de; 26 Translational and Clinical Research Institute, Faculty of Medical Sciences, Newcastle University, Newcastle Upon Tyne NE2 4HH, UK; 27 Algarve Biomedical Center Research Institute (ABC-Ri), Algarve Biomedical Center (ABC), Faculdade de Medicina e Ciências Biométicas, University of Algarve, 8005-139 Faro, Portugal; 28 Nuffield Department of Clinical Neurosciences, Oxford Parkinson’s Disease Center, University of Oxford, Oxford OX3 9DU, UK; laura.parkkinen@ndcn.ox.ac.uk; 29 Clinical Ageing Research Unit, Newcastle University, Campus for Ageing and Vitality, Newcastle Upon Tyne NE4 5PL, UK; 30 Neurosciences, Newcastle Upon Tyne NHS Foundation Trust, Newcastle Upon Tyne NE1 4LP, UK; 31 Department of Medical and Surgical Sciences, Institute of Neurology, Magna Graecia University, 88100 Catanzaro, Italy; an.quattrone@unicz.it; 32 Health, Psychology and Communities Research Centre, Department of Psychology, Manchester Metropolitan University, Manchester M15 6BH, UK; n.ray@mmu.ac.uk; 33 Department of Neurology, University of Würzburg, 97080 Würzburg, Germany; reich_m1@ukw.de; 34 Applied Neuroscience Research Group, Brain and Mind Research Programme, Central European Institute of Technology, Masaryk University, 625 00 Brno, Czech Republic; irena.rektorova@fnusa.cz; 35 Movement Disorders Centre, First Department of Neurology, St. Anne’s University Hospital, Faculty of Medicine, and CEITEC, Masaryk University in Brno, 625 00 Brno, Czech Republic; 36 Edmond J. Safra Parkinson Disease Program, Morton and Gloria Shulman Movement Disorder Unit, Krembil Brain Institute, University Health Network, University of Toronto, Toronto, ON M5T 2S8, Canada; antonio.strafella@uhn.ca; 37 Institute of Medical Science, Temerty Faculty of Medicine, University of Toronto, Toronto, ON M5S 1A8, Canada; 38 Brain Health Imaging Centre, Campbell Family Mental Health Research Institute, Centre for Addiction and Mental Health, University of Toronto, Toronto, ON M6J 1H4, Canada; 39 Fondazione IRCCS Istituto Neurologico Carlo Besta, 20133 Milan, Italy; fabrizio.tagliavini@istituto-besta.it; 40 Department of Neurology, University Hospital and Medical Faculty, University of Cologne, 50937 Cologne, Germany

**Keywords:** Parkinson’s disease biomarkers, multimodal neuroimaging, Proteinopathies, co-pathology, prodromal and atypical parkinsonism, translational precision medicine

## Abstract

The “Neuroimaging and Pathology Biomarkers in Parkinson’s Disease” course held on 12–13 September 2025 in Milan, Italy, convened an international faculty to review state-of-the-art biomarkers spanning neurotransmitter dysfunction, protein pathology and clinical translation. Here, we synthesize the four themed sessions and highlights convergent messages for diagnosis, stratification and trial design. The first session focused on neuroimaging markers of neurotransmitter dysfunction, highlighting how positron emission tomography (PET), single photon emission computed tomography (SPECT), and magnetic resonance imaging (MRI) provided complementary insights into dopaminergic, noradrenergic, cholinergic and serotonergic dysfunction. The second session addressed in vivo imaging of protein pathology, presenting recent advances in PET ligands targeting α-synuclein, progress in four-repeat tau imaging for progressive supranuclear palsy and corticobasal syndromes, and the prognostic relevance of amyloid imaging in the context of mixed pathologies. Imaging of neuroinflammation captures inflammatory processes in vivo and helps study pathophysiological effects. The third session bridged pathology and disease mechanisms, covering the biology of α-synuclein and emerging therapeutic strategies, the clinical potential of seed amplification assays and skin biopsy, the impact of co-pathologies on disease expression, and the “brain-first” versus “body-first” model of pathological spread. Finally, the fourth session addressed disease progression and clinical translation, focusing on imaging predictors of phenoconversion from prodromal to clinically overt stages of synucleinopathies, concepts of neural reserve and compensation, imaging correlates of cognitive impairment, and MRI approaches for atypical parkinsonism. Biomarker-informed pharmacological, infusion-based, and surgical strategies, including network-guided and adaptive deep brain stimulation, were discussed as examples of how multimodal biomarkers may inform personalized management. Across all sessions, the need for harmonization, longitudinal validation, and pathology-confirmed outcome measures was consistently emphasized as essential for advancing biomarker qualification in multicentre research and clinical practice.

## 1. Introduction

Despite major advances in symptomatic treatment, Parkinson’s disease (PD) and atypical parkinsonian syndromes remain characterized by substantial unmet needs in diagnosis, individual trajectories, and disease modification. Current clinical criteria still rely predominantly on motor manifestations that emerge only after extensive neurodegeneration, while early and prodromal stages remain difficult to identify with sufficient biological certainty. Moreover, marked clinical heterogeneity, frequent co-pathologies, and variable trajectories of motor and cognitive decline limit the precision of both patient counseling and clinical trial design. In routine practice, clinicians face persistent challenges in differentiating PD from atypical parkinsonism, predicting the conversion to clinically manifest synucleinopathy (i.e., development of parkinsonism and/or dementia fulfilling diagnostic criteria) in individuals with high-risk states (hereafter referred to as “phenoconversion”), such as isolated/idiopathic REM sleep behavior disorder (iRBD), and anticipating individual response or vulnerability to advanced therapies.

In parallel, research efforts have been hindered by the lack of validated, pathology-anchored biomarkers capable of capturing the multisystem nature of parkinsonian disorders. Dopaminergic imaging alone is insufficient to explain non-motor symptoms, cognitive impairment, or treatment variability, while emerging disease-modifying strategies increasingly demand biologically defined cohorts and robust markers of target engagement. Against this background, the first edition of the “Neuroimaging and Pathology Biomarkers in Parkinson’s disease” held on 12–13 September 2025 in Milan, Italy, was designed to integrate neuroimaging, molecular pathology, and clinical modeling across the full disease continuum. The meeting was conceived as a comprehensive exploration of biomarkers that define, track, and guide the treatment of PD, with a strong emphasis on both neurotransmitter imaging and neuropathological correlates. Across four structured sections, the program led participants through an integrative journey, moving from molecular mechanisms to clinical applications. A practical, scenario-based workflow for biomarker selection is summarized at the end of the manuscript. All biomarkers discussed in this report, including their target, modality, disease stage, clinical or research application, and level of maturity, are summarized in Supplementary Table S1. Authors contributions are summarized in Supplementary Table S2.

## 2. Imaging Neurotransmitter Dysfunction in PD

### 2.1. Dopaminergic Dysfunction

*Molecular Imaging*. Dopaminergic dysfunction remains the central hallmark of PD, with neuroimaging playing a pivotal role in both diagnosis and research. Molecular imaging studies have employed various radiotracers to probe different elements of the presynaptic dopaminergic system. Among these, [^18^F]DOPA PET traces the uptake and conversion of levodopa to dopamine (DA) via aromatic amino acid decarboxylase (AADC), which becomes upregulated as a compensatory mechanism in early disease stages [1]. In contrast, dopamine transporter (DAT) SPECT imaging offers a more sensitive assessment in prodromal and early PD stages [1,2]. This imaging modality is now considered the gold standard for visualizing nigrostriatal degeneration in vivo, achieving diagnostic accuracies approaching 95% [3]. It is particularly useful in detecting at-risk individuals, such as those with iRBD or hyposmia [4,5]. However, there may be external interference, vascular insults or compressive factors such as normal pressure hydrocephalus, in which the reduction in tracer uptake does not necessarily correspond to degeneration but to mechanical-ischemic causes [6,7,8]. Interpretation must account for compensatory downregulation of DAT and upregulation of AADC in early PD, which may cause DAT imaging to overestimate and F-DOPA PET to underestimate the actual neuronal loss [1,2]. These observations are especially pronounced in younger patients and correlate with earlier development of motor fluctuations and dyskinesias [2].

Post-synaptic dopaminergic receptors, particularly D2 receptors, can also be evaluated using specific PET ligands. The dopaminergic synapse can be studied in its postsynaptic compartment using tracers that bind to D2 dopaminergic receptors. In principle, in early-stage PD there is no alteration in the density of postsynaptic receptors, unlike in atypical parkinsonism, where degeneration affects not only the substantia nigra but also the striatal compartment. However, during the course of the disease, also in relation to the use of dopaminergic drugs in PD, a reduction in receptor density can be observed, also due to internalisation phenomena. Furthermore, it should be considered that, as these are transmembrane surface proteins, they undergo continuous dynamic phenomena, so the main problem is the reproducibility of the results. However, the study of postsynaptic receptors can be useful for assessing dopamine reserves: if a dopaminergic challenge is performed using a drug that increases dopamine release (e.g., methamphetamine or levodopa) or a psycho-cognitive stimulus [9,10] that releases dopamine, the baseline and post-challenge study of dopamine receptor density provides an accurate estimate of dopamine release, and it is estimated that a 10% reduction in receptors corresponds to a fivefold increase in dopamine release.

**Figure 1:**
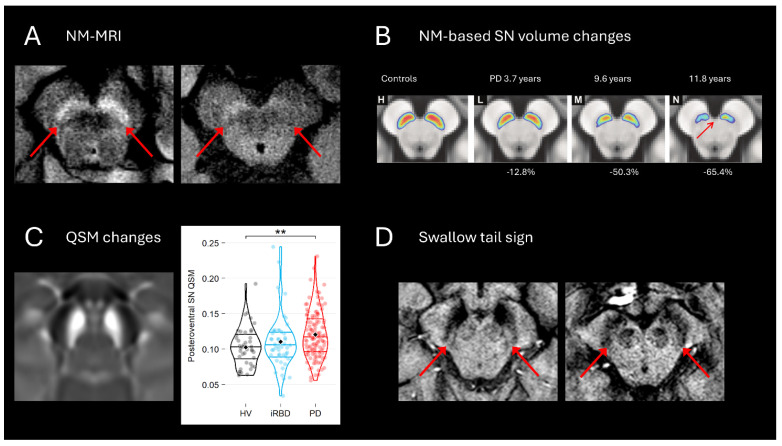
**MRI of Dopaminergic System in PD**. (**A**). NM-sensitive MRI axial slice passing by the midbrain showing the high signal intensity of the substantia nigra (red arrows) in a healthy subject (**image on the left**) and a patient with PD (**image on the right**). (**B**). Probability maps of the substantia nigra using NM signal intensity overlaid a brain template in a group of healthy subjects (controls) and patients with PD with 3.7-, 9.6- and 11.8-years disease duration. Percentages indicate the percent reduction in substantia nigra volume for each disease duration (adapted from Biondetti E. et al. Brain 2020 [13]). (**C**). (**Left**): Quantitative susceptibility map of the substantia nigra in the axial plane. (**Right**): Violin plots comparing the baseline distributions of QSM values in the posteroventral nigra between a group of healthy subjects (black), patients with isolated RBD (blue) and PD (red) (**, statistically significant difference) adapted from Gaurav R, et al. [20]). (**D**). (**Left image**): High-resolution axial 3D echo planar images showing the area of high signal intensity in the inferolateral part of the substantia nigra in a healthy control (swallow tail sign, red arrows). (**Right image**): This sign is not visible in a patient with PD).

*Magnetic Resonance Imaging*. Recent advances in MRI-based biomarkers complement molecular imaging by capturing dopaminergic neurodegeneration non-invasively. Neuromelanin-sensitive MRI (NM-MRI) detects the loss of neuromelanin signal in the substantia nigra (SN), particularly in its posterolateral regions corresponding to motor territories (Figure 1A). These changes are detected about 5 to 6 years in the SN before the diagnosis of PD, whereas dopaminergic dysfunction using radiotracers is detected about 10 years before onset [11]. NM-MRI can categorize PD patients from controls by visual reading with an accuracy of about 85% after training of the rater [12]. NM signal changes are: (a) spatially selective: NM-sensitive MRI signal loss progresses anatomically from sensorimotor to associative and limbic SN subregions [13] (Figure 1B); (b) early: volume and signal changes are detected in prodromal stages such as iRBD and LRRK2 asymptomatic mutation carriers [14]; (c) correlated with markers of striatal dysfunction using DAT-SPECT and (d) with motor, cognitive and behavioral scores [13]; (e) detected in all neurodegenerative parkinsonism and therefore cannot distinguish between PD and atypical parkinsonism [14]. Iron-sensitive MRI, especially quantitative susceptibility mapping (QSM), detects iron accumulation in the SN pars compacta and reveals changes up to 10 years before diagnosis [15,16] (Figure 1C). Iron increases in the nigrosome-1 region, a PD-vulnerable substructure, are detected in patients with iRBD and correlate with motor and cognitive impairment in PD [17]. Loss of the “swallow tail sign” on susceptibility-weighted imaging indicates nigrosome-1 degeneration, now a key radiological signature of PD [18] (Figure 1D). Nigrosome-1 overlaps with the swallow tail sign but nigrosome 1 (detected in histology) and the swallow tail sign (MRI sign) are not identical [19]. MRI-histological comparative studies have shown that the dominant contribution to iron-sensitive techniques in nigrosome-1 is iron accumulated in the neuromelanin of dopaminergic neurons, providing a multi-parametric MRI framework for early and specific PD detection [16,17,18,19].

### 2.2. Noradrenergic Dysfunction

The noradrenergic (NA) system, long overshadowed by dopaminergic mechanisms, is now recognized as a crucial contributor to PD pathology, particularly to its non-motor features, which include sleep disruption, cognitive impairment, mood disorders and autonomic symptoms [21]. Autopsy studies consistently demonstrate extensive degeneration of the locus coeruleus (LC), sometimes even exceeding nigral neuronal loss [22]. Cortical NA innervation is also profoundly reduced, with corresponding decreases in NA levels across frontal, limbic, and thalamic structures [23]. These findings underpin the hypothesis that LC degeneration may represent an upstream event in PD pathophysiology, supporting staging models of prodromal neurodegeneration (e.g., “Body-first” subtype hypothesis described below).

**Figure 2:**
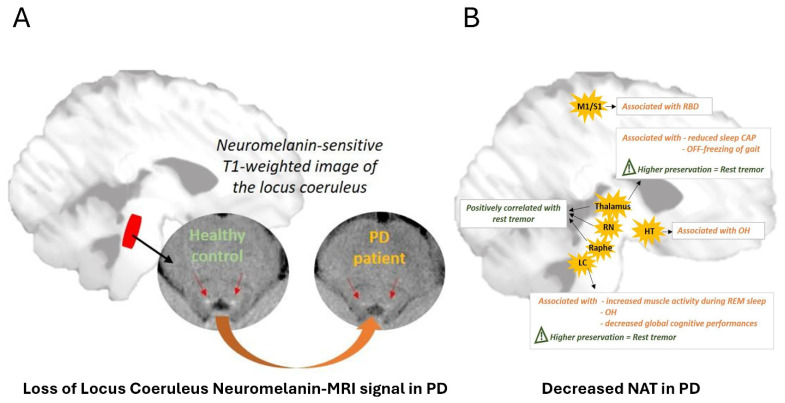
**MRI and Molecular Imaging of Noradrenergic System in PD**. (**A**). NM-sensitive MRI sagittal representation of the Locus Coeruleus. Neuromelanin signal is reduced in patients with PD (**image on the right**) compared to healthy subjects (**image on the left**). (**B**). Molecular imaging of the NAT: areas of reduced updated and their association with motor and nonmotor clinical features (e.g., rest tremor, freezing of gait; sleep, orthostatic hypotension, cognitive dysfunction) are depicted.

In vivo imaging methods now allow to probe these processes directly. At the molecular level, [^11^C]MeNER PET imaging of the noradrenaline transporter (NAT) has shown widespread reductions in the thalamus, frontal cortex, and insula, without direct correlation to disease duration, indicating that NA dysfunction occurs early and progresses non-linearly [24]. Reduced [^11^C]MeNER binding has been associated with the presence of sleep disorders, orthostatic hypotension, freezing of gait and cognitive impairment [25]. Recently, [^11^C]yohimbine PET targeting α2-adrenergic receptors (α2-ARs) has shown reduced binding in motor cortex, thalamus, insula, and putamen. Clinically, reductions in thalamic α2-ARs correlated with tremor, while reduced binding in limbic regions was associated with anxiety symptoms [26]. NM-MRI of the LC demonstrates reduced signal in PD, and these changes correlate with iRBD, depression, apathy, and cognitive dysfunction [27,28] (Figure 2).

Taken together, these findings highlight that (i) LC–NA dysfunction may be a robust biomarker for prodromal and early PD, complementing DAT imaging and clinical evaluation, and (ii) targeting the NA system holds promise for alleviating non-motor symptoms. Combining multimodal imaging with clinical phenotyping should refine sensitive, specific NA biomarkers to identify at-risk individuals, track progression, and support NA-based therapeutic trials.

### 2.3. Cholinergic Dysfunction

**Figure 3:**
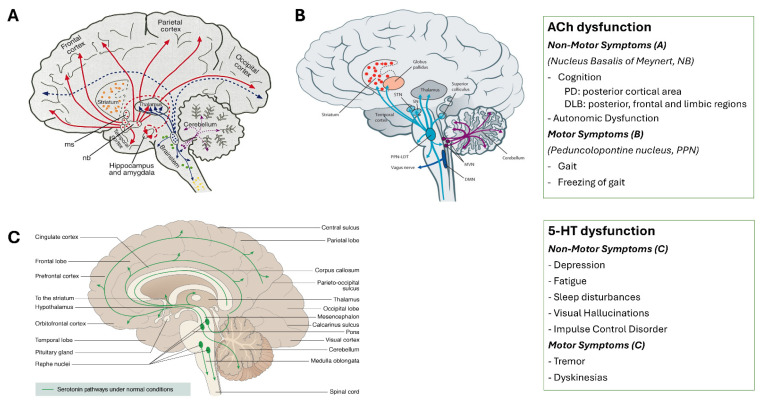
**Cholinergic and Serotonergic Pathways in Parkinson’s Disease and Dementia with Lewy bodies**. (**A**). Cholinergic pathways originating from the nucleus basalis of Meynert (NB) project to widespread cortical regions and are crucial for cognitive processing. These patterns are associated with progressive cognitive impairment and may also contribute to autonomic dysfunction. (**B**). Brainstem cholinergic pathways arising from the pedunculopontine nucleus (PPN) innervate subcortical motor structures and spinal locomotor circuits. (Adapted from Bohnen NI et al. [50]). (**C**). Serotonergic pathways. Ascending projections from the raphe nuclei modulate a wide range of non-motor symptoms (including depression [43], fatigue [47], sleep disturbances [48], visual hallucinations [46], and impulse control disorders [49]) and contribute to motor features (e.g., tremor [44] and levodopa-induced dyskinesias [45]), reflecting the interplay between 5HT and DA systems in PD pathophysiology (Modified from https://neurotorium.org/image/nt-serotonin-pw-normal/, accessed on 7 January 2026).

The brain receives cholinergic projections from several basal forebrain and brainstem nuclei. The peripheral autonomic nervous system is composed of numerous cholinergic projections, such as preganglionic parasympathetic vagal neurons innervating internal organs. Cholinergic pathways, both central and peripheral, are profoundly affected in Lewy body diseases (LBD), with broad implications for cognition, gait, and autonomic function [29] (Figure 3A,B).

*Molecular Imaging.* PET imaging quantifies presynaptic cholinergic terminals either indirectly, by measuring acetylcholinesterase (AChE) density ([^11^C]-donepezil) or activity ([^11^C]PMP), or directly, by assessing vesicular acetylcholine transporter density ([^18^F]FEOBV PET). [^18^F]FEOBV PET has revealed region-specific cholinergic loss in PD, forming a reproducible pattern [30]. In early PD, degeneration is confined to posterior cortical areas, whereas in dementia with Lewy bodies (DLB), loss extends to frontal and limbic regions, paralleling clinical progression toward dementia [31] (Figure 3A). These patterns align with *post-mortem* findings that identify hippocampal Lewy pathology and cholinergic depletion as correlates of dementia [32]. In prodromal PD (iRBD), [^11^C]-donepezil PET has shown cortical AChE reductions, limited to posterior areas in cognitively intact individuals. Those with mild cognitive impairment additionally show frontal reductions [33]. Importantly, AChE decline tracks with cognitive deterioration and may predict phenoconversion [34]. Peripheral cholinergic loss is also observable. PET studies show degeneration in the colon, pancreas, and small intestine of patients with PD, DLB, and iRBD [35]. The most severe cholinergic loss has been reported in PD patients with prodromal iRBD, supporting the notion that these patients represent a “body-first” subtype of PD, in which pathology initially arises in the peripheral autonomic nervous system [35,36]. Future cholinergic PET studies should assess the longitudinal cholinergic changes across the LBD spectrum. Given the strong relationship with cognitive decline and dementia, cholinergic imaging methods could serve as outcome-measure in clinical trials with focus on preserving cognitive function in LBD.

*Magnetic Resonance Imaging.* Volumetric MRI of the cholinergic basal forebrain (cBF), including the nucleus basalis of Meynert, reveals that atrophy in this region predicts progression of cognitive impairment in PD, independent of dopaminergic loss [37]. This structural degeneration also correlates with levodopa-unresponsive gait impairment, indicating a shared cholinergic contribution to both motor and cognitive symptoms [38] (Figure 3A,B). Multimodal studies integrating MRI with [^18^F]FEOBV PET demonstrate that reduced cBF volume corresponds with decreased cholinergic terminal integrity, validating MRI-derived cBF atrophy as a biomarker of presynaptic cholinergic denervation [39]. In parallel, diffusion-weighted imaging of the pedunculopontine nucleus (PPN) shows that elevated free water content predicts subsequent gait deterioration in PD [40]. Although these diffusion metrics are not yet clinically sensitive or specific, they offer insights into brainstem microstructural degeneration. Integrating these MRI measures with intraoperative electrophysiology, higher PPN free water has been associated with altered modulation of subthalamic nucleus beta oscillations after levodopa administration [41]. This suggests that PPN pathology may influence DA-mediated motor circuit dynamics (Figure 3B). Collectively, these imaging findings provide strong evidence that MRI can non-invasively capture region-specific cholinergic degeneration relevant to symptom progression in PD.

### 2.4. Serotonergic Dysfunction

The serotonergic system contributes significantly to both motor and non-motor symptoms of PD. Lewy pathology and neuronal loss in raphe nuclei, along with reduced serotonin (5HT) levels in the caudate nucleus, hypothalamus, hippocampus, and frontal cortex have been documented [42].

Tremor has been particularly linked to serotonergic alterations. Reduced 5HT transporter (SERT) availability in the raphe nuclei, as measured by [^123^I]FP-CIT SPECT, negatively correlates with tremor severity [43]. Likewise, PET imaging with [^11^C]WAY-100635 shows lower 5-HT_1A_ receptor binding in tremor-dominant PD [44]. Levodopa-induced dyskinesias (LIDs) involve serotonergic terminals that convert and release DA aberrantly. Dual PET studies with [^11^C]DASB and [^11^C]Raclopride confirm this maladaptive dopaminergic release [45], underscoring the intricate interplay between dopamine and serotonin systems in PD. On the non-motor side, serotonergic deficits are associated with visual hallucinations, depression, fatigue, sleep disturbances, and impulse control disorders (ICDs) [46,47,48,49] (Figure 3C). Recent work suggests that serotonin contributes to the chronic maintenance of compulsive behaviors in ICDs, distinct from dopaminergic initiation [49].

## 3. Molecular Imaging of Pathology

### 3.1. Imaging α-Synuclein Pathology

Although α-Synuclain (aSyn) is a 140-amino-acid presynaptic protein thought to contribute to synaptic plasticity, its physiological role is still incompletely defined; what is clear is that its pathological aggregation is central to PD, DLB, and multiple system atrophy (MSA), and therefore a primary target for disease-modifying strategies. In parallel with the rapid expansion of α-Syn-directed immunotherapies, the field has faced a persistent bottleneck: the lack of a validated α-Syn PET biomarker for subject selection and target engagement, meaning that most trials still depend largely on clinical diagnostic labels.

**Figure 4:**
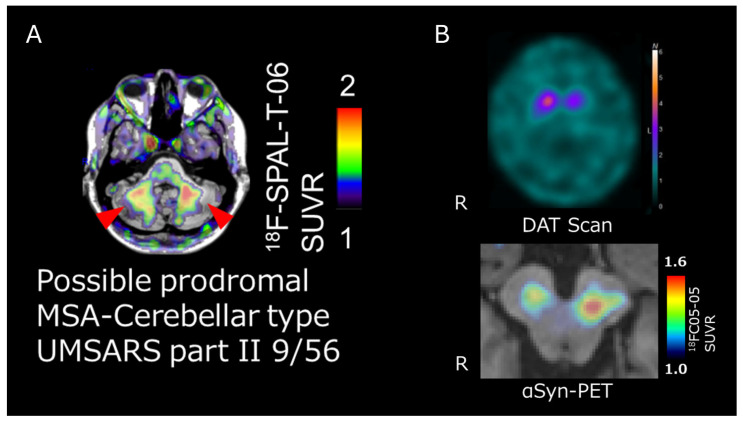
**α-Synuclein PET imaging in vivo**. (**A**). Axial SUVR PET image of [^18^F]SPAL-T-06 overlaid on MRI in a patient with possible prodromal multiple system atrophy of cerebellar type (MSA-C). Shown at the level of the middle cerebellar peduncles, the tracer accumulates in regions characteristic of MSA (the pons, middle cerebellar peduncles, and cerebellar white matter), achieving high-contrast visualization of α-synuclein pathology (red arrowheads). (**B**) Concordance between striatal DAT-SPECT uptake reduction (upper panel) and [^18^F]-C05-05 accumulation in the midbrain (lower panel), both showing left-predominant changes contralateral to the right-dominant motor symptoms, in a patient with familial PD due to the A53T mutation in the SNCA gene.

The development of α-Syn PET ligands faces multiple challenges, including the low abundance of α-Syn aggregates (especially at earlier disease stages), reliance on β-sheet targeting, intracellular localization, post-translational modifications, and structural heterogeneity between in vivo and in vitro conditions. Compared with amyloid-β (Aβ), the in vivo concentration of α-Syn aggregates is dramatically lower, forcing tracer development into a narrow window where exceptionally high affinity and selectivity are required. Biochemical work has estimated α-Syn levels in DLB brain tissue in the range of 0.1–0.25 μg/g, whereas Aβ in Alzheimer’s disease (AD) can be 100–200 μg/g, a several-hundred-fold difference in target abundance that directly translates into signal-to-noise challenges for PET ligands [51]. Additional obstacles include reliance on β-sheet binding (with a high risk of cross-reactivity), intracellular localization, post-translational modifications, and structural heterogeneity between in vitro fibrils and the conformers found in human disease. Against this backdrop, Tagai and colleagues highlighted an instructive translational pathway: leveraging expertise from tau PET development. [^18^F]PM-PBB3 (florzolotau) is a tracer enabling high-contrast visualization of tau pathology in both AD and non-AD tauopathies [52]. Then, [^18^F]SPAL-T-06 was introduced as a novel tracer with high binding affinity for α-Syn aggregates. In clinical imaging, [^18^F]SPAL-T-06 produced high-contrast signal in MSA, with uptake in the basal ganglia, midbrain, pons, cerebellar peduncles and deep cerebellar white matter, regions consistent with MSA pathology [53]. Particularly compelling for biomarker utility, expanded datasets suggested detectability within one year of MSA onset, including patients suspected to be in a prodromal MSA-C stage (Figure 4A), implying potential for very early, pathology-driven stratification [*unpublished data*].

In PD and DLB, where α-Syn deposition is thought to be present in lower abundance, another novel tracer, [^18^F]-C05-05, successfully visualized α-Syn pathology. Tracer accumulation in the midbrain correlated with motor severity in PD, supporting the concept that α-Syn PET might evolve from a diagnostic adjunct into a progression or severity biomarker—provided performance is robust across cohorts and scanners [54]. In a proof-of-concept translational extension, PET scanning in familial PD due to the A53T mutation in the *SNCA* gene showed concordance between tracer accumulation, DAT-SPECT uptake reduction, and the symptomatic body side, suggesting a potential role in linking molecular pathology to functional DAergic impairment at the individual level [*unpublished data*] (Figure 4B, with kind referral from Dr. Eriko Igami, Dr. Shin-ichi Ueno and Professor Taku Hatano, Juntendo University) [^18^F]C05-05 appears to carry relatively higher background signal, compromising contrast compared with [^18^F]SPAL-T-06 in MSA [54,55]. Conversely, [^18^F]SPAL-T-06 and [^18^F]ACI-12589 have not yet reliably visualized Lewy bodies in PD or DLB [55]. Moreover, image-pathology correlation has not yet been established for any of the currently available α-Syn PET ligands.

For the development of next-generation aSyn PET tracers, essential requirements include sub-nanomolar affinity, rapid brain clearance to enable dynamic imaging, and optimized lipophilicity to reduce background signal. Achieving these properties, as emphasized in recent studies, will be critical to advance the clinical feasibility of aSyn imaging [56].

In conclusion, α-Syn imaging is only beginning to show practical feasibility. Initial human studies with [^18^F]-SPAL-T-06 and [^18^F]-C05-05 provide encouraging evidence toward enabling objective diagnosis, patient stratification, and therapeutic evaluation in synucleinopathies.

### 3.2. Imaging Tau Pathology

Progressive supranuclear palsy (PSP) and corticobasal degeneration (CBD) are primary 4-repeat (4R) tauopathies, with straight tau filaments accumulating in neurons and glia and producing characteristic clinical syndromes [57]. Paired-helical filament tau co-pathology in Lewy body disorders contributes to heterogeneity and influences disease progression [58]. Tau PET has expanded understanding of these mixed-pathology states [59,60]. These findings highlight how tau PET provides biologically relevant information even in predominantly synuclein-driven disorders.

Second-generation ligands were developed specifically to detect non-AD tau. Florzolotau (PM-PBB3) shows robust PSP uptake with distribution patterns approximating the known topography of subcortical and brainstem involvement [52]. However, the binding may be relatively unspecific, since in MSA of the parkinsonian subtype, florzolotau PET shows basal ganglia uptake as well [61]. This is where tracer comparison and mechanistic validation become essential. Autoradiography studies provided strong mechanistic evidence for tracer specificity of PI-2620, whereas ligands such as MK-6240 and RO-948 show limited binding in 4R tauopathies [62]. These findings align with in vivo data in multicenter evaluations of the German Imaging Initiative for 4R Tauopathies (GII4T): PI-2620 PET consistently detects elevated subcortical signal in PSP and cortical–subcortical involvement in CBD, with good correspondence to clinical severity [63,64,65,66]. Beyond structural mapping, tau PET also relates to functional alterations. In PSP, subcortical tau accumulation is associated with reductions in frontal and limbic cortical perfusion, suggesting that tau pathology contributes to distributed network dysfunction [65]. The interpretation of regional PET signal is guided by detailed *post-mortem* studies delineating cell-type specific hierarchical tau progression across brainstem, basal ganglia, frontal cortex and cerebellar structures [67]. In vivo tau PET signal in tauopathies corresponds primarily to neuronal and oligodendroglial tau accumulation, rather than astroglial lesions [68]. This cell-type specificity clarifies how PET signal should be biologically interpreted in PSP and CBD [68]. In corticobasal syndrome (CBS), PI-2620 PET identifies amyloid-positive (AD pathology) and 4R-tauopathies, enabling the detection of underlying pathology in this heterogeneous group [69].

Despite these advances, key challenges limit immediate clinical translation. Specificity for 4R tau is not uniform across ligands; off-target binding remains a concern; and no tau PET tracer has yet undergone phase III validation to establish diagnostic accuracy. Standardized multicenter protocols as well as improved kinetic modeling and autopsy-confirmed cohorts will be essential. Nevertheless, tau PET is emerging as a promising biomarker for early diagnosis, disease stratification and future 4R tau-targeted therapeutic trials.

### 3.3. Imaging Amyloid and Co-Pathology

The advent of PET tracers for amyloid has profoundly transformed both research and clinical practice in AD, enabling in vivo visualization of β-amyloid (Aβ) plaques. The field’s transformation began with the [^11^C]PiB (Pittsburgh Compound-B), the first amyloid radiopharmaceutical derived from Thioflavin-T, but clinical scalability accelerated after validation and approval of multiple [^18^F]-labeled tracers (with longer half-life and broader access), enabling amyloid PET to move beyond a handful of research centers [70]. Contemporary reviews emphasize amyloid PET’s role in improving diagnostic accuracy across neurodegenerative disorders and in clarifying atypical or early-onset cognitive phenotypes, including mild cognitive impairment (MCI) and preclinical stages [71]. Overall concordance between amyloid PET and cerebrospinal fluid (CSF) measures is high (often 85–90%), yet PET provides a distinct advantage: topographic localization of plaque burden, which CSF cannot deliver and which can become crucial in ambiguous cases and in research stratification [72]. Furthermore, the recent introduction of quantitative approaches, such as standardized uptake value ratios (SUVR), the centiloid scale, and deep learning–based tools, yielded robust, reproducible, and clinically validated measures of amyloid burden.

Mixed pathology, characterized by the coexistence of AD and PD, is frequently observed, particularly among elderly patients with PD. This overlap involves β-amyloid plaques, tau neurofibrillary tangles, and aSyn Lewy bodies, with significant implications for clinical symptomatology and disease progression. Autopsy studies indicate that 30–40% of patients diagnosed with PD, especially those with cognitive decline, also exhibit substantial AD pathology [52]. The presence of AD co-pathology in PD is associated with accelerated cognitive deterioration, earlier onset of dementia, and increased risk of hallucinations, although clinical recognition remains challenging [73]. Amyloid PET has proven effective in detecting such co-pathology: in autopsy-confirmed cases, positive amyloid PET scans corresponded to the presence of amyloid plaques, whereas negative scans aligned with the absence of significant amyloid pathology. Moreover, amyloid PET reliably differentiates dementia with Lewy bodies (DLB) from AD and mixed DLB/AD pathology. Lower amyloid PET uptake is characteristic of pure DLB, whereas higher uptake indicates AD or mixed pathology, with diagnostic accuracy reaching up to 93%. In DLB, elevated amyloid PET signals predominantly reflect diffuse Aβ plaques, while in AD, neuritic plaques predominate. This distinction is critical for accurate in vivo identification of AD co-pathology in DLB [74].

### 3.4. Imaging Neuroinflammation

Microglia make up about 5–10% of brain cells playing a central role in innate immunity. When the central nervous system (CNS) is disrupted by injury or disease, microglia transition from a resting to an activated state, releasing cytokines, reactive oxygen species, and other immune mediators. Activated microglia can adopt either a pro-inflammatory or anti-inflammatory phenotype, depending on the context and stage of disease, influencing whether inflammation leads to tissue damage or repair [74]. PET targeting microglia enables an in vivo assessment of neuroinflammation using radiopharmaceuticals that bind to biomarkers of activation, primarily the translocator protein 18-kDa (TSPO). TSPO is minimally expressed in resting microglia but markedly upregulated during neuroinflammatory responses. Its increased expression in activated microglia, astrocytes, and infiltrating macrophages makes it a reliable biomarker for neuroinflammation and a valuable tool for studying disease progression and therapeutic response [75]. Over the past two decades, multiple generations of TSPO ligands have been developed for PET imaging. The first-generation ligand, [^11^C]-PK11195, remains widely used but has limitations such as a short half-life, low brain penetration, and high non-specific binding. To improve signal quality, second-generation ligands (e.g., [^11^C]-PBR28, [^18^F]-DPA-714, [^18^F]-FEPPA) were introduced, but their clinical use is complicated by sensitivity to the rs6971 polymorphism, which affects binding affinity and introduces variability in imaging results. This led to the development of third-generation tracers (e.g., [^18^F]-GE180, [^11^C]-ER176), designed to minimize genetic variability and improve diagnostic reliability [75].

Multiple studies have demonstrated that microglial activation plays a central role in the pathogenesis of PD. PET imaging studies using the first-generation TSPO ligand [^11^C]-PK11195 have consistently shown increased microglial activation in PD [74]. Two early studies reported higher PK binding in the striatum and SNc of PD patients compared to healthy controls [76,77]. Voxel-based PET analyses have expanded these findings revealing that neuroinflammation extends beyond the nigrostriatal system. Increased PK binding was detected in the frontal and temporal areas [78], while widespread microglial activation was observed in temporal, parietal, and occipital regions [79]. In PD patients with dementia, activation extended further to cingulate, striatal, and frontal regions, overlapping with areas of glucose hypometabolism. Studies using second-generation TSPO ligands (e.g., [^11^C]-DPA-713, [^18^F]-FEPPA) have provided mixed results. Elevated [^11^C]-DPA-713 binding was found in occipital, temporal, and parietal cortices, with further increases after one year, though limited by small sample size and modeling constraints [80]. Two [^18^F]-FEPPA studies showed genotype-dependent differences in binding but no consistent overexpression of TSPO in PD or correlation with disease severity or duration [81,82].

## 4. From Pathology to Mechanisms and Models

### 4.1. aSyn Biology: From Aggregates to Therapeutic Strategies

Building on the molecular imaging advances discussed earlier, this section broadened the lens to the biology of PD and related synucleinopathies, emphasizing why disease-modifying therapies have repeatedly failed despite aSyn centrality to neuropathology. Lessons from AD, where only a few therapeutic approaches such as lecanemab, and donanemab have shown modest benefit, highlight the difficulties in targeting complex neurodegenerative processes, and suggest we should account for biological heterogeneity, timing of intervention, and target engagement, rather than assuming a single mechanism or therapy will be uniformly effective across patients. [83,84]. In PD, trial failures have been linked to patient heterogeneity, inadequate dosing and timing, limited blood–brain barrier penetration, and insufficient proof of target engagement [84], all set against a still-incomplete understanding of the molecular basis of disease [85]. Within this framework, aSyn remains the most coherent biological anchor. Genetic evidence (*SNCA* gene mutations, multiplications, and common variants) supports its causal relevance for disease onset and progression [86]. Yet how aSyn drives degeneration remains debated, with two non-exclusive hypotheses: a toxic gain-of-function via aggregation and seeding, and a loss-of-function through sequestration or “synucleinopenia.” Most likely, a combination of these two hypotheses is involved in the disruption of neuronal circuit integrity and, ultimately, in cell death. Yet fundamental questions remain unresolved: Does aggregation itself cause disease? Does it impair the physiological functions of aSyn? Or does toxicity arise from specific oligomeric intermediates?

The process of aSyn aggregation is thought to be a multistep and heterogeneous process whereby distinct assemblies, ranging from oligomers to fibrils, exhibit diverse structural and toxic properties. Recent technological advances have enabled new insights into the complexity of the aggregation process. Super-resolution microscopy revealed morphological diversity of inclusions, including atypical “ring-like” structures distinct from classical Lewy bodies [87]. Expansion microscopy further demonstrated that these distinct inclusion types coexist within the same cellular environment. Complementary structural studies using cryo-electron microscopy have shown how disease-linked mutations alter fibril structure and stability, thereby providing a molecular explanation for unusual neuropathological phenotypes observed in affected patients.

These mechanistic insights were directly connected to biomarker and therapeutic development. A major translational leap has been the rise of ultrasensitive methods capable of detecting misfolded aSyn seeds (see below), enabling earlier -and potentially biologically cleaner-trial populations [88]. Therapeutic pipelines were reviewed across passive/active immunotherapies, antisense oligonucleotides to reduce aSyn expression, and small molecules aimed at reducing aggregation or improving proteostasis; while safety has generally been encouraging, robust demonstrations of target engagement and clinical benefit remain elusive [89]. To address biological heterogeneity, classification frameworks such as the *SynNeurGe* research diagnostic criteria were presented as key enablers for future trials, providing a structure to move from symptom-based enrollment to biology-driven stratification [90]. Taken together, these advances illustrate how aSyn biology connects genetics, neuropathology, molecular biophysics, and clinical translation. Elucidating the diversity of aSyn assemblies, refining disease classification frameworks, and improving patient stratification will be critical steps toward the development of effective disease-modifying therapies for PD and related disorders.

### 4.2. aSyn Seed Amplification Assays: Present and Future

A recurring theme across the course was the need to anchor diagnosis to biology while patients are still early enough to benefit from intervention. Definitive diagnosis of synucleinopathies still rests on neuropathology [91], but seed amplification assays (SAAs) were presented as a practical bridge from *post-mortem* certainty to in vivo decision-making. SAAs replicate in vitro the misfolding/aggregation kinetics of disease proteins and can detect misfolded aSyn in a broad range of samples, including CSF, skin, olfactory mucosa, saliva, blood, and gastrointestinal tissue [92].

In CSF, multiple studies report high diagnostic accuracy for PD, DLB, and MSA, supporting real-world incorporation. Notably, SAA has already been adopted as a supportive biomarker for MSA in the Movement Disorder Society diagnostic criteria, an important milestone for a technology still in rapid evolution [93]. Beyond “positive/negative” classification, one of the most compelling translational promises is the ability of SAA to discriminate aSyn strains, aligning biological readouts with distinct clinicopathological entities and enabling precision-medicine strategies [94,95,96]. This strain-specific recognition opens the door to precision medicine approaches, moving beyond the “one-size-fits-all” therapeutic paradigm. Complementing CSF work, serum-based detection of propagative aSyn seeds has further reinforced the feasibility of peripheral signatures, potentially widening access to biologically anchored diagnosis [96].

Prodromal detection was emphasized as the most transformative application. Importantly, SAA has shown the ability to detect αSyn pathology in various biological samples during prodromal stages, including individuals with iRBD [97,98,99]. Longitudinal studies show that SAA positivity in iRBD is strongly associated with phenoconversion to PD or DLB, often occurring years before clinical diagnosis [100]. This early detection capability positions SAA as a powerful tool for risk stratification and for enabling potential early intervention. Remarkably, beyond classical synucleinopathies, SAA detected aSyn co-pathology in other diseases, including AD and amyotrophic lateral sclerosis (ALS), suggesting broader implications of αSyn misfolding across neurodegenerative diseases [101,102].

Despite its promise, SAA faces challenges including the lack of standardized protocols, the need for specialized infrastructure, and limited disease specificity, as positivity has also been observed in other neurodegenerative conditions. While not a standalone diagnostic tool, SAA serves as a valuable indicator of synucleinopathy processes, supporting early detection, risk stratification, and personalized care when used alongside clinical and other diagnostic data.

### 4.3. The Role Skin Biopsy in the Diagnosis of Parkinsonism

Over three decades, skin biopsy has become a core method for assessing peripheral nervous system disorders and, more recently, for demonstrating peripheral involvement in diseases traditionally considered CNS-predominant [103]. In PD, early work demonstrated an asymmetric loss of intraepidermal nerve fibers (IENF) associated with sensory impairment [103]. Subsequent studies expanded this concept, showing that cutaneous denervation involves small and large fibers, Meissner corpuscles and myelinated endings, and autonomic fibers; critically, these changes were described as intrinsic to PD, present early, largely independent of levodopa exposure, and most evident on the clinically more affected side [104].

Longitudinal data strengthened the biological plausibility of these findings: IENF loss correlates with severity and duration and progressively spreads to the contralateral side, reducing early asymmetry. Moreover, peripheral neuroplasticity appears stage-dependent: IENF regeneration was observed after rehabilitation in patients with shorter disease duration and milder motor impairment, but not in more advanced disease, suggesting peripheral repair capacity declines alongside neurodegenerative progression [105]. In atypical parkinsonism, skin biopsy profiles may further support differential diagnosis: PSP showed more severe length-dependent denervation than PD and correlated with severity [106], while MSA demonstrated clear postganglionic sudomotor denervation, challenging older assumptions of purely preganglionic involvement [107,108]. In an early-disease comparison, combined morpho-functional sudomotor evaluation produced a composite sudomotor parameter with discriminative utility for MSA vs. PD [109].

Beyond denervation, cutaneous phosphorylated aSyn (p-syn) detection has become a disease-specific biomarker. After the first *post-mortem* observation of p-syn in PD skin in the context of brain Lewy pathology, multiple groups have demonstrated in vivo detection across PD, MSA, DLB, and pure autonomic failure using antibodies to phosphorylated aSyn [110,111,112,113,114]. Importantly, p-syn positivity has also been reported in iRBD, supporting its potential role as a prodromal biomarker preceding motor onset [115]. Although current evidence demonstrates high sensitivity and specificity of this biomarker in distinguishing synucleinopathies from controls and from non-synucleinopathies [112,113,114] the standardization of methodological parameters—including biopsy site, section thickness, staining protocol, and antibody selection—remains essential before its routine application in clinical practice.

### 4.4. The Role of Co-Pathologies in PD: The Neuropathologist’s Perspective

A key conceptual consolidation of the section was the idea that co-pathology is the rule rather than the exception. While PD, Parkinson’s disease dementia (PDD), and DLB are clinically distinct, they are pathologically identical, characterized by widespread Lewy bodies (LBs) and neuronal loss in selective nuclei such as the SN. Neuropathological studies show that a large fraction of PD cases also meet criteria for AD and frequently harbor additional tauopathies, cerebrovascular lesions, and cerebral amyloid angiopathy [116]. Tau pathology is found in nearly all PD cases to some degree and becomes more extensive in PDD and DLB, where Braak tau stages 5–6 and neocortical amyloid deposition are common [117]. In parallel, amyloid plaque burden tends to be highest in DLB, intermediate in PDD, and lowest in PD, mapping onto clinical gradients of cognitive vulnerability [118].

Clinically, the course emphasized that dementia risk in PD correlates more strongly with Aβ and tau burden than with cortical Lewy body density, and that combined pathology shortens time-to-dementia across cohorts [119,120]. Mechanistically, mixed pathology appears non-random: tau and aSyn can co-localize in the same neurons [121], and double-labeling studies across cortical and limbic regions demonstrate partial overlap of aSyn, tau, and Aβ, supporting molecular cross-seeding rather than chance co-aggregation [122]. TDP-43 adds a further layer: classically linked to ALS and frontotemporal dementia (FTD), TDP-43 inclusions appear in 7% of PD, 19% of PDD, and over 30% of DLB cases with AD pathology, forming an additional pathological layer that may exacerbate neurodegeneration [123]. Mechanistic studies suggest that aSyn, tau, and amyloid-β interact synergistically. Misfolded monomers promote each other’s aggregation, share degradation pathways, and trigger microglial activation and neuroinflammation, creating a self-perpetuating cycle of damage [119]. Genetic susceptibility, via LRRK2, MAPT, ApoE, or rare TDP-43 mutations, may further influence these relationships. Even cerebrovascular pathology is frequently observed in PD, though its contribution to cognitive impairment remains debated [124].

Understanding these overlapping pathologies is crucial for therapy development and patient stratification. Most biomarker research has focused on detecting aSyn in CSF or peripheral tissues, but this must expand to include tau, amyloid-β, TDP-43, and vascular markers. Early results show that plasma p-tau181 may differentiate PD from controls, although longitudinal studies suggest limited correlation with progression [125]. Future advances in AI-assisted quantitative neuropathology and spatial omics will enable precise mapping of multiple protein aggregates within single neurons and their microenvironments. Such integrated molecular pathology promises to uncover why some patients develop dementia while others do not, and to guide the design of targeted, disease-modifying therapies that address not only aSyn but its pathological partners.

### 4.5. Brain-First vs. Body-First: A Model to Reconcile Clinical, Imaging, and Pathological Heterogeneity

To integrate these biological and neuropathological complexities with patient-level heterogeneity [126], the “*brain-first*” vs. “*body-first*” framework was proposed as a unifying model. The model was positioned as a response to limitations of traditional symptom-based subtyping and staging systems, which often fail to align clinical phenotypes with post-mortem spreading patterns [127,128,129].

Two assumptions were emphasized: (1) intracellular aSyn aggregation is a key driver of degeneration, interacting with oxidative stress, mitochondrial dysfunction, and lysosomal impairment; and (2) pathology typically begins in a single site and spreads trans-synaptically through the connectome [130]. From this, two dominant trajectories emerge: (i) a *brain-first* subtype, where the pathology starts unilaterally in the brain, most likely in the olfactory bulb or amygdala based on neuropathological studies [131], and (ii) a *body-first* subtype, where the pathology initiates in the peripheral autonomic nervous system, and subsequently spreads to the sympathetic trunk and brainstem and from there ascends to the rest of the brain. Since iRBD is caused by neuronal dysfunction in pontine nuclei [132], located inferior to the SN, this is considered a strong marker of the body-first subtype.

Support for the model comes from neuropathology, machine learning, and multimodal imaging [133]. Post-mortem work has described divergent gradients consistent with brain-first vs. body-first profiles [134], and these patterns have been reproduced using a machine-learning algorithm (SuStaIn) designed to predict progression patterns from cross-sectional data, which defined two distinct body-first subtypes with predominance in either the sympathetic or parasympathetic nervous system [131]. Molecular imaging studies further support the existence of these two subtypes: body-first PD patients (defined by pre-motor iRBD), show much more severe cardiac sympathetic and colon parasympathetic degeneration compared to brain-first PD (without iRBD) [36]. Large de novo cohorts similarly link cardiac sympathetic denervation to iRBD, orthostatic hypotension, and constipation [135]. iRBD cohorts exhibited marked cardiac sympathetic denervation before overt nigrostriatal dopaminergic degeneration, strongly suggesting that the neurodegenerative process starts in the periphery and later spreads to the brain [136]. Conversely, sequential MIBG imaging showing initially preserved cardiac sympathetic innervation that becomes impaired later supports the existence of a brain-first trajectory [137]. Finally, emerging peripheral aSyn detection aligns with the model’s predictions: distinct skin aSyn distribution patterns have been reported in PD with versus without premotor iRBD (i.e., body-first and brain-first), with body-first showing distal-to-proximal gradients and higher positivity, and brain-first showing a proximal-to-distal pattern [138]. In summary, the brain-first and body-first model is a framework to understand the heterogenous nature of LBD and it is supported by numerous neuropathological, imaging, and clinical studies. However, more studies are needed to further explore and validate the existence of these subtypes.

## 5. Tracking Disease Progression

### 5.1. From Prodromal to Overt Clinical Stages

How can we identify and quantify neurodegeneration when classic clinical syndromes (parkinsonism, dementia) are not yet fully expressed? Within emerging biological frameworks, both the integrated staging proposal for neuronal aSyn disease and the *SynNeurGe* research criteria, prodromal stages start being considered legitimate targets for stratification and trials [90,139]. The most established clinical biomarkers of the prodromal stage are iRBD, hyposmia, and orthostatic hypotension with iRBD representing the most studied clinical gateway to future PD, DLB, or MSA [140]. *Post-mortem* evidence confirms that iRBD can already harbor aSyn pathology in REM-atonia brainstem structures even before in-life phenoconversion [141] and more than 90% of iRBD patients show in vivo evidence of aSyn pathology in the CSF and in the skin [142]. Therefore, it has been suggested that most iRBD patients, with an adult onset and without clear brain lesions that may cause iRBD, might be more appropriately named iRBD due to synucleinopathy, instead of being defined isolated or idiopathic [143].

From an imaging standpoint, the course emphasized that the core objective is not simply “early diagnosis,” but measurable neurodegeneration suitable for individual risk estimation and as a therapeutic trial endpoint. The most established tool remains DAT SPECT [90,139]. The largest longitudinal study on iRBD patients showed that visually assessed DAT-SPECT is among the best predictors of phenoconversion (hazard ratio, HR 1.98) [140] and semi-quantified DAT-SPECT combined with clinical metrics achieved substantially stronger risk prediction (hazard ratios reported up to ~5–6) [144], highlighting that the analysis pipeline can be as important as the modality itself. Critically, moving from group inference to individual applicability, the “most affected putamen” has emerged as a particularly informative DAT-SPECT feature that characterizes iRBD due to synucleinopathy and enriches for short-term converters [5].

Brain [^18^F]FDG-PET is the second most promising neuroimaging technique in synucleinopathies, as it can track progression through disease-related metabolic networks, such as the PD-related pattern [90,145]. In iRBD, the iRBD-related metabolic pattern has been reported to resemble early PD physiology [146], and newer patterns (including a conversion-related pattern and PD-with-iRBD-related pattern) extend this logic toward phenoconversion forecasting [147,148]. Particularly compelling, longitudinal network-change studies suggest that [^18^F]-FDG-PET network expression may evolve systematically as individuals transition from prodromal states into overt synucleinopathy, supporting [^18^F]-FDG-PET as a candidate progression biomarker rather than a mere diagnostic adjunct [149]. Together, DAT-SPECT and [^18^F]-FDG-PET were framed as a pragmatic two-pronged approach: one anchored in nigrostriatal terminal integrity, the other capturing distributed network-level consequences of neurodegeneration.

Current literature data encourage the use of these approaches in disease-modifying trials, both for stratification purposes (e.g., identifying iRBD patients at high risk of short-term phenoconversion) and for objectively monitoring longitudinal neurodegeneration.

### 5.2. The Concept of Motor and Cognitive Resilience vs. Compensation in Neurodegenerative Disorders: Two Sides of the Same Coin?

Since the late 1990s, it has been evident that some individuals remain clinically unaffected despite marked pathology, while others decline rapidly. This variability has led to the introduction of reserve (cognitive, brain, and more recently motor reserve) and compensation to explain individual differences, with motor reserve gaining particular relevance in PD [150].

Reserve mechanisms are often grouped under the umbrella of *resilience*, referring to processes that allow coping with the effects of neurodegeneration. Brain reserve reflects a more passive form, rooted in structural integrity, while *cognitive reserve* denotes a dynamic process enabling adaptive strategies to maintain function. Analogously, *motor reserve* refers to the preservation of motor performance despite dopaminergic degeneration in PD, likely mediated by adaptations of neural networks. Neuroimaging studies indicate that higher motor reserve is associated with greater regional functional connectivity, increased grey matter volume in motor-related regions, and enhanced serotonergic innervation, suggesting these mechanisms mitigate disease burden [151,152]. Lifestyle moderators were emphasized as plausible “drivers” of reserve: educational attainment has been associated with higher network attack tolerance in attention systems, and lifetime physical activity may preserve motor function by moderating relationships between network robustness and clinical performance [153].

Unarguably, compensation contributes to resilience, but its extent likely reflects individual resilience capacity. For instance, motor compensation has been associated with increased M1–striatal connectivity and interhemispheric cortical plasticity [154]. Whether such mechanisms occur broadly across patients or represent resilience in a subset of individuals, which may be influenced by moderator variables remains unclear. Moreover, PD itself may induce maladaptive compensatory changes, highlighting the need for carefully designed longitudinal studies.

Crucially, resilience represents stable protective capacity built-up over life, whereas compensation may rather relate to short-term network adaptations. Distinguishing between the two is therefore essential for understanding disease heterogeneity and differences in clinical trajectories. The implication for progression tracking is subtle but crucial as imaging signals may reflect (i) accumulating pathology, (ii) compensatory reconfiguration, or (iii) resilience capacity built across the lifespan. Disentangling these components is essential if imaging endpoints are to be interpreted correctly in trials. A deeper understanding of these processes will support the development of personalized interventions that leverage education, physical activity, and targeted therapies to enhance resilience and delay onset and clinical decline in PD.

### 5.3. Network-Driven Conceptualization of PD

Beyond regional atrophy and focal microstructural changes, MRI-based connectomics offers a framework to conceptualize PD as a disorder of large-scale brain networks rather than isolated structures. Structural and functional connectome analyses consistently show that hubs within the basal ganglia–thalamo–cortical loops and fronto-parietal control network represent critical nodes of vulnerability, where early dopaminergic degeneration and misfolded protein spread converge [155]. In drug naïve and early PD, graph-theoretical metrics derived from resting-state fMRI and diffusion MRI reveal a reorganization of brain architecture within basal ganglia circuits and between these regions and sensorimotor, occipital, and associative cortices, in line with a “brain dys-connectome” model of disease propagation [156].

Network-level signatures are already detectable at prodromal stages. In subjects with iRBD, widespread connectivity changes across motor and extra-motor regions have been described, supporting the concept that network failure may precede nigrostriatal neuronal loss [157]. Longitudinal functional MRI studies indicate that progressive disruption of dorsal attention and sensorimotor networks differentiates iRBD converters from non-converters, with changes in striato-cortical coupling predicting phenoconversion to PD or DLB over time [149]. These findings align with the body-first versus brain-first framework, in which intrinsic network topology constrains the routes of aSyn propagation and shapes the timing and distribution of clinical manifestations. This is also supported by recent evidence showing that in manifest PD structural and functional links may act as conduits for “disease exposure” across the connectome [158].

Connectome-derived measures have been linked to both motor and cognitive trajectories in PD. Altered connectivity between basal ganglia and sensorimotor, frontal, parietal, and occipital regions predicts the development of treatment-related motor complications [159], while distributed network changes in temporal and occipital hubs are associated with faster cognitive decline [160].

Together, these data indicate that MRI-based connectomics can bridge molecular pathology, regional imaging markers, and clinical heterogeneity, providing routes of progression, as well as substrates for staging, risk stratification, and individualized trial design in neurodegenerative disorders.

### 5.4. MRI for Tracking Cognitive Impairment in Lewy Body Diseases

Structural, diffusion and functional MRI can be used to detect, predict and monitor cognitive impairment in LBD, like PD and DLB. In prodromal DLB, cortical thinning has been reported in insular, anterior cingulate, and medial frontal regions [161], while an occipital atrophy “signature” may offer superior sensitivity to distinguish prodromal DLB from healthy aging [162]. Beyond single-region markers, clustering approaches are increasingly used to define biologically meaningful subtypes: data-driven MRI clustering in probable DLB with over a 3-year follow-up period has identified three subgroups with distinct atrophy distributions and cognitive trajectories: 1. cortical atrophy predominant subtype with faster cognitive decline (including older-aged individuals with higher load of white matter hyperintensities); 2. frontal-occipital cortical atrophy “intermediate” subtype with regard to cognitive decline; 3. subcortical atrophy predominant and cognitively stable subtype (including younger individuals with higher frequency of cognitive fluctuations) [163]. Similar logic is now being applied in PD, where longitudinal deformation-based morphometry has differentiated clinical–neuroanatomical trajectories over multi-year follow-up, with “diffuse-malignant” profiles showing accelerated atrophy in regions such as the precuneus, temporal and fusiform gyri, and cerebellum alongside faster cognitive decline [164].

A central emphasis was the cholinergic system. MRI markers of nucleus basalis of Meynert (NBM) integrity have been repeatedly linked to cognitive impairment in PD and may predict future decline [165]. Yet white-matter connectivity appears even more sensitive: reduced integrity of NBM projection pathways and broader cholinergic white-matter tracts were strongly associated with cognition and may serve as an earlier biomarker of dementia risk across the Lewy body continuum [166,167]. Microstructural techniques extend this further. Free-water imaging has been proposed as a marker of microstructural deterioration, and thalamic dorsomedial nucleus free water correlates with cognitive decline in PD [168,169]. Longitudinal free-water studies in DLB similarly report change in regions relevant to visuospatial processing, motor function, and cholinergic networks, with associations to evolving cognitive and motor scores over two-year follow-up [170].

Noradrenergic integrity was also featured via NM-MRI. In mild cognitive impairment with Lewy bodies, decreased NM signal in the caudal right LC distinguished patients from controls and related to visual memory performance, with associations reported as independent of AD-related co-pathology by plasma biomarkers [171]. In PD, degeneration of LC and SNc showed selective cognitive associations, reinforcing a multi-nuclei model of cognitive impairment rather than a cortex-only narrative [172].

Finally, resting-state fMRI work illustrates how functional connectivity may capture both decline and compensation: cognitive scores in PD have been linked to reduced fronto-parietal network connectivity and altered coupling with default mode systems [173], while in prodromal DLB increased dorsal striatal connectivity to temporo-parietal regions may reflect compensatory recruitment supporting executive functions [174]. Longitudinal rs-fMRI studies further suggest that different patterns of connectivity change relate to cognitive stability versus decline across follow-up, underscoring the need for time-aware interpretation [175].

Overall, advanced MRI enables early detection and monitoring of cognitive impairment in Lewy body diseases by capturing macrostructural and microstructural changes, particularly within cholinergic pathways, and by characterizing large-scale brain connectivity.

### 5.5. MRI Biomarkers in Atypical Parkinsonian Disorders

A consistent message in the atypical parkinsonism segment was that biomarker value depends on context of use and availability, not simply performance in expert centers. This is particularly important given global variability in access to advanced neuroimaging infrastructure: the field must balance innovation with scalable, low-cost approaches [176].

Routine structural MRI remains highly informative, especially in PSP, where characteristic atrophy patterns can guide differential diagnosis [177]. Machine learning and deep learning approaches using structural MRI are promising in clinical settings: automated categorization of parkinsonian syndromes has shown feasibility [178], and automated differentiation work continues to expand in larger-scale, clinically relevant cohorts [179]. There are three families of structural MRI markers:

1. Volumetric measures are stable, robust, and particularly effective for tracking progression—often outperforming clinical scales as trial endpoints in PSP or MSA [180,181]. Yet volumes have not consistently predicted future progression at the individual level, limiting their use as prognostic biomarkers despite their strength as progression biomarkers [182].
2. Planimetric indices targeting the most affected regions (e.g., midbrain area, pons-to-midbrain ratio, MR Parkinsonism Index (MRPI and MRPI 2.0)) provide strong diagnostic discrimination for PSP and have been linked to future PSP-specific features and underlying PSP pathology [183,184]. These measures showed excellent classification performance in supporting the differential diagnosis between PSP and other parkinsonism in several studies and meta-analyses, also demonstrating usefulness in predicting future development of PSP specific features and PSP pathology [179,185]. However, their use in routine radiological workflows is limited by time/expertise requirements.
3. This gap motivated the call for simple linear measures, potentially deployable by technicians during acquisition or by clinicians, provided that standardized measurement procedures, anatomical landmarks, and validation in large international cohorts can be established [176]. In short, the field is converging on a pragmatic stratification: volumetry for progression tracking in trials, planimetry for diagnostic discrimination in expert settings, and linear measures as the candidate bridge to widespread clinical implementation, ideally augmented by AI tools as they mature.

### 5.6. Management of PD: Pharmacological and Surgical Approaches

*Oral and infusion pharmacological approaches.* Therapy optimization in PD increasingly requires moving beyond intermittent, oral dopaminergic replacement toward approaches that reduce pharmacokinetic variability, address non-motor burden, and tailor advanced interventions to individual risk profiles. Oral levodopa remains the cornerstone of symptomatic treatment, yet it has a short plasma half-life and a restricted intestinal absorption window with competitive transport, so that only a small proportion of each dose reaches the brain; these limitations are amplified by common gastrointestinal dysfunction in PD [186]. Together with progressive nigrostriatal degeneration, pulsatile dopaminergic stimulation contributes to motor fluctuations and levodopa-induced dyskinesias, providing the rationale for continuous dopaminergic strategies [187]. Consequently, over the past decades, substantial efforts have focused on developing strategies to improve levodopa delivery to the brain and to achieve more continuous DA receptor stimulation. Three major approaches have been pursued: (1) management of gastrointestinal dysfunction; (2) early use of enzymatic inhibitors; and (3) implementation of continuous drug-delivery systems.

A frequently underestimated determinant of treatment response is the gastrointestinal tract, which is a pharmacokinetic and pathophysiological bottleneck of response to levodopa. Specific gut bacteria, such as *Enterococcus faecalis*, harbor tyrosine decarboxylase enzymes capable of converting levodopa to dopamine in the periphery, thereby reducing drug availability for central transport [188,189]. An interspecies pathway for levodopa metabolism has been described, supporting the concept that microbial enzymatic networks—rather than single taxa—may drive clinically relevant drug degradation and side effects, and can potentially be inhibited pharmacologically [190].

Another strategy aiming at stabilizing levodopa plasma levels is the early use of monoamine oxidase B inhibitors (iMAO-B) and catechol-O-methyltransferase inhibitors (iCOMT) inhibitors in combination with AADC inhibitors. By reducing peripheral levodopa metabolism, COMT inhibitors increase levodopa bioavailability and prolong its plasma half-life. Early initiation of COMT inhibition has been proposed to prevent or delay the onset of motor fluctuations, thereby supporting a more continuous dopaminergic stimulation early in the disease course [182].

When oral therapy fails to satisfactorily compensate motor and non-motor fluctuations, continuous drug-delivery systems represent a major advance in the management of advanced PD. Levodopa–carbidopa intestinal gel (LCIG) infusion provides continuous intestinal levodopa delivery and has demonstrated robust long-term efficacy in reducing motor fluctuations and dyskinesias [191]. However, its invasive nature and device-related complications limit its widespread use. More recently, foslevodopa/foscarbidopa subcutaneous infusion has emerged as a less invasive alternative, with promising short-term efficacy and safety data. Although long-term outcomes are still under investigation, this approach may significantly expand access to continuous levodopa therapy [191]. Further innovation includes the development of levodopa–carbidopa–entacapone gel infusion, which requires lower infusion volumes, enabling smaller pumps and improved patient usability, potentially enhancing adherence and quality of life [190]. Continuous drug delivery can also be achieved using subcutaneous apomorphine infusion. Recent studies have demonstrated that night-time apomorphine infusion alone significantly improves sleep disturbances in patients with PD, highlighting its therapeutic role beyond motor symptom control [192].

*Surgical approaches*. The adoption of deep brain stimulation (DBS) for PD has steadily increased over the past decades and it is now an established treatment, along with other advanced options: infusion therapies and MRI-guided focused ultrasound (MRgFUS). In fact, the introduction of MRgFUS has increased the pool of patients referred for functional neurosurgery—including DBS. The recent introduction of sensing-capable devices has expanded our understanding of brain physiology in PD and open the field to adaptive DBS (aDBS), now approved in most countries worldwide. Local field potentials (LFPs) are reliable and feasible biomarkers of patients’ clinical states, reflecting a range of conditions including misplacement of leads implanted for DBS, motor fluctuations, and the quality and quantity of sleep [193]. While the beta band (13–30 Hz) is the classic LFP of interest, newer studies have explored other frequencies, particularly the finely tuned gamma band as a marker of overtreatment [194]. Neuroimaging advancements have also informed targeting and programming of these patients (as outlined below), although a clear workflow embedding sensing, neuroimaging and clinical features is still missing. A major unmet need is precision patient selection for STN-DBS. Although it has been established that levodopa responsiveness predicts short-term motor benefit, longer-term trajectories diverge substantially, likely reflecting underlying biology (including genetic background) and comorbidities such as cerebrovascular burden [195]. Evidence from monogenic PD suggests gene-specific differences in outcomes after STN stimulation (Table 1) [196], and broader frameworks (called ‘surgicogenomics’ in analogy with pharmacogenomic) propose integrating CNS-expressed genetic variation beyond classic PD genes to refine selection and counseling [197]. In parallel, objective predictors are emerging from network-level functional MRI and PET imaging and microstructural MRI, which may inform outcome prediction and risk stratification [198,199]. Lastly, fluid biomarkers are slowly gaining attention (Table 2).

*Neuroimaging approaches in DBS*. DBS has become an established therapy for PD, dystonia, obsessive–compulsive disorder (OCD), and other movement and psychiatric disorders. While the clinical efficacy of DBS is well documented, optimizing targeting and programming remains a major challenge. Here, recent advances in imaging in DBS targeting, computational modeling, and connectomics that aim to translate neuroimaging into individualized, data-driven treatment strategies are presented. While electrode placement has traditionally relied on landmarks, network mapping shows that therapeutic effects depend on engaging disease-relevant pathways. Different DBS targets, including the subthalamic nucleus used for PD and OCD, converge on specific cortical–subcortical circuits, underscoring that efficacy reflects network modulation rather than stimulation of a single nucleus [204]. A major conceptual advance is the shift from “where the lead is” to “which circuit is modulated,” leveraging structural and functional connectomics to interpret interindividual variability and to understand why nominally similar implants can yield divergent outcomes [204,205,206,207,208,209]. Computational modeling further supports this transition. Combining postoperative imaging with estimates of the stimulated volume allows creation of outcome maps that predict motor benefit. In PD, probabilistic mapping has identified subregions of the subthalamic nucleus linked to gait improvement [205]. These methods also enable rapid “in silico” exploration of stimulation parameters, compressing what would require lengthy manual testing into minutes, and early artificial intelligence–assisted tools such as C-Surf have improved programming efficiency and short-term outcomes in dystonia [206]. Connectomics extends this approach by mapping stimulation sites onto distributed structural and functional networks. Imaging also informs safety: a network associated with cognitive decline under STN stimulation enabling avoidance of high-risk contacts has been identified [207]. Finally, integrating functional MRI and adaptive paradigms may accelerate personalized, closed-loop DBS by capturing frequency-dependent network effects in vivo [208,209].

## 6. Conclusions

**Figure 5:**
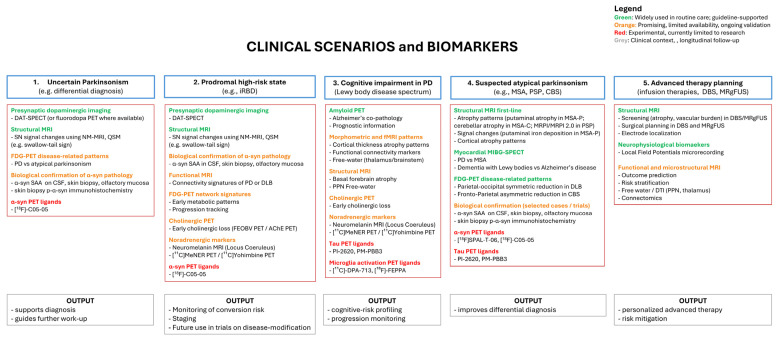
Practical workflow for imaging and biomarker selection in PD and parkinsonism. The flowchart links common clinical scenarios (uncertain parkinsonism, prodromal high-risk states, cognitive impairment, suspected atypical parkinsonism, and advanced-therapy planning) to a stepwise selection of established and emerging biomarkers across neurotransmitter dysfunction imaging, pathology imaging, and peripheral/CSF assays. To facilitate implementation and to highlight research priorities for biomarker qualification, tools are color-coded by maturity level: clinically established (green) vs. emerging, ongoing validation (orange) vs. experimental and limited to research (red).

The first edition of the “Neuroimaging and Pathology Biomarkers in Parkinson’s Disease” course delivered a uniquely comprehensive program spanning the full translational arc of parkinsonian disorders: from multisystem neurotransmitter dysfunction and advanced magnetic resonance and molecular imaging, to in vivo assessment of protein aggregation, neuroinflammation, and co-pathologies anchored in neuropathological frameworks. By integrating imaging and pathology-based perspectives, the faculty highlighted convergent mechanisms that explain clinical heterogeneity, refine prodromal and differential diagnosis, and support biology-driven stratification for trials. Finally, the program also connected pathophysiology to practice, outlining biomarker-informed pharmacological, infusion, and surgical strategies, toward personalized management and more efficient disease-modifying studies. A practical, scenario-based workflow for biomarker selection is summarized in Figure 5.

## Figures and Tables

**Table 1:** Systematic review of PD-associated gene and outcome of STN DBS (from Kuusimaki T et al. [196]).

Gene	Cases Reported (Detailed)	Target	Unfavorable Motor Outcome
*LRRK2*	87 (73)	STN: 79, NA: 8	10 (13.7%)
*PRKN*	67 (57)	STN: 51, GPi: 5, Zi: 1, NA: 10	6 (10.5%)
*GBA1*	50 (30)	STN: 33, GPi: 4, VIM: 1, NA: 12	12 (40%)
*SNCA*	5 (5)	STN: 4, GPi: 1	0
*VPS35*	5 (5)	STN: 3, NA: 2	1 (20%)
*PINK1*	5 (5)	STN: 4, GPi: 1	1 (20%)

**Table 2:** Plasmatic biomarker and DBS.

Reference	Aim	Biomarkers	Meaning
Carrillo et al., 2024 [200]	Discriminant	Acylcarnitine, Sphingolipids, fatty acid oxidation, steroids, leptin, TNFα, GFAP, BDNF, etc.	Different from HC, drug-naïve and patients on L-dopa
Frank et al. 2025 [201]	Discriminant	GFAP, NfL	Higher after surgery, especially in cognitive impaired pts (GFAP)
Gong et al., 2023 [202]	Predictor	Bleomycin hydrolase and Creatine kinase M-type	Downregulated in responders
Zhou et al., 2022 * [203]	Predictor	CRP, NfL, S100β	Higher in POD (CSF in particular)

*: also CSF; POD: post-operative delirium.

## Data Availability

No new data were created or analyzed in this study. Data sharing is not applicable to this article. The present manuscript is based on the lectures delivered during the “Neuroimaging and Pathology Biomarkers in Parkinson’s Disease” course, held on 12–13 September 2025 in Milan, Italy. Access to the original lecture materials is restricted to course participants.

## Supplementary Materials

The following supporting information can be downloaded at: https://www.mdpi.com/article/10.3390/brainsci16010110/s1, Table S1: Summary of biomarkers in Parkinson’s disease, dementia with Lewy bodies, and atypical parkinsonism; Table S2: Faculty contributions.

## Abbreviations

4R, 4-repeat; 5HT, serotonin; AADC, aromatic L-amino acid decarbox-ylase; AChE, acetylcholinesterase; AD, Alzheimer’s disease; aDBS, adaptive deep brain stimulation; ALS, amyotrophic lateral sclerosis; aSyn, α-Synuclein; Aβ, amyloid-β; CBD, corticobasal degeneration; cBF, cholinergic basal forebrain; CBS, corticobasal syndrome; CNS; central nervous system; CSF, cerebrospinal fluid; DA, dopamine; DAT, dopamine transporter; DLB, dementia with Lewy bodies; FDG, fluorodeoxyglucose; FTD, frontotem-poral dementia; ICDs, impulse control disorders; iCOMT, catechol-O-methyltransferase inhibitors; IENF; intraepidermal nerve fibers; iMAO-B, monoamine oxidase B inhibitors; iRBD, isolated/idiopathic REM sleep behavior disorder; LBD, Lewy body diseases; LBs, Lewy bodies; LC, locus coeruleus; LCIG, Levodopa–carbidopa intestinal gel; LFP, local field potential; LIDs, Levodopa-induced dyskinesias; MCI, mild cognitive impairment; MRgFUS, MRI-guided focused ultrasound; MRI, Magnetic Resonance Imaging; MRPI, Magnetic Resonance Parkinsonism Index; MSA, multiple system atrophy; NA, Noradrena-line; NAT, noradrenaline transporter; NBM, nucleus basalis of Meynert; NM-MRI, Neuro-melanin-sensitive MRI; OCD, obsessive–compulsive disorder; OH, orthostatic hypoten-sion; PD, Parkinson’s disease; PDD; Parkinson’s disease dementia; PET, positron emission tomography; PPN, pedunculopontine nucleus; PSP, Progressive supranuclear palsy; p-syn, phosphorylated aSyn; QSM, quantitative susceptibility mapping; SAA, seed amplifi-cation assay; SERT, serotonin transporter; SN, substantia nigra; SPECT, single photon emission computed tomography; SUVR, standardized uptake value ratios; TSPO, trans-locator protein 18-kDa.
