# Supplementary material for: Neuroimaging and Pathology Biomarkers in Parkinson’s Disease and Parkinsonism
Source: Brain Sci. 2026 Jan 19;16(1):110. doi: 10.3390/brainsci16010110 (PMC12838680; doi:10.3390/brainsci16010110)
Supplement: Supplementary file 1 [file brainsci-16-00110-s001.zip › brainsci-4090154-supplementary.pdf]

Supplementary Table S1. Summary of biomarkers in Parkinson's disease, dementia with Lewy bodies, and atypical parkinsonism. Biomarkers are grouped by domain, modality, disease stage, clinical or research use, and maturity level. Color codes indicate clinical readiness: clinically established (green); emerging with/withour ongoing validation (orange); experimental, limited to research (red).

Domain / Target	Modality	Disease Stage	Clinical and/or Research Use	Maturity Level
**IMAGING NEUROTRANSMITTER DYSFUNCTION**				
**DOPAMINE**				
**Dopamine (presynaptic)**	DAT-SPECT / [^{18}F]DOPA PET	Prodromal, Early	Diagnosis. Trial enrichment	🟢 Clinical
**Dopamine (postsynaptic)**	D2 PET	Advanced	Post-synatpic dopamine function	🔴 Research
**Neuromelanin in SN**	NM-MRI	Prodromal, Early	Supportive early marker	🟠 Emerging (Ongoing validation)
**Nigral Iron Deposition**	QSM / SWI (MRI)	Prodromal, Early	Early detection. Diagnosis	🟢 Clinical
**NORDADRENALINE**				
**Noradrenaline (NAT)**	[^{11}C]MeNER PET	Prodromal, Early	Non-motor phenotyping	🟠 Emerging
**Noradrenaline (LC)**	LC NM-MRI	Prodromal, Early	Non-motor phenotyping	🟠 Emerging (Ongoing validation)
**ACETYLCOLINE**				
**Cholinergic System (NBM, PPN)**	[^{11}C]-Donepezil, [^{18}F]FEOBV PET	Early - Advanced	Cognition, gait dysfunction	🟠 Emerging
**Cholinergic System (NBM)**	Volumetric MRI	Early - Advanced	Predicting cognitive decline	🟠 Emerging
**SEROTONIN**				
**Serotonin (Raphe, SERT)**	[^{123}I]FP-CIT, [^{11}C]WAY-100635 PET	Variable	Tremor, psychiatric symptoms	🟠 Emerging
**IMAGING BRAIN PATHOLOGY**				
**α-Synuclein Aggregates**	α-syn PET ([^{18}F]C05-05, SPAL-T-06)	Early. Research	Diagnosis, patient stratification	🔴 Research

**Domain / Target**	**Modality**	**Disease Stage**	**Clinical and/or Research Use**	**Maturity Level**
**Tau (4R)**	PET (PI-2620, PM-PBB3)	Atypical PKS	Differential diagnosis	🟠 Emerging
**β-Amyloid**	PET ([^{11}C]PiB, [^{18}F]Florbetaben)	DLB, PDD	Diagnosis. Prognosis. Co-pathology	🟢 Clinical
**Neuroinflammation**	TSPO ([^{11}C]PK11195, [^{18}F]DPA-714)	Variable	Pathophysiological research	🔴 Research
**NETWORK AND OTHER IMAGING MARKERS**				
**Glucose Hypometabolism**	FDG-PET	All stages	Differential diagnosis, staging.	🟢 Clinical
**Structural MRI (Morphometry)**	MRI	All stages	Diagnosis of PSP, MSA, CBS	🟢 Clinical
**Free Water Imaging**	Diffusion MRI	Early	Gait, cognition prediction	🟠 Emerging
**DBS Planning**	Structural MRI	Advanced	Personalized therapy planning (DBS)	🟢 Clinical
**Functional Connectivity**	fMRI, DTI, Connectomics	Advanced	Personalized therapy planning (DBS, MRgFUS)	🟠 Emerging
**IN VIVO PATHOLOGY AND NEUROPHYSIOLOGICAL MARKERS**				
**Seed Amplification Assay (SAA)**	CSF (± skin biopsy, olfactory mucosa, blood)	Prodromal, Early	Biological confirmation of synuclein pathology. Application in trials	🟠 Emerging (ongoing validation)
**Skin Biopsy (p-α-syn detection)**	Immunofluorescence	Early - Advanced	Synucleinopathy diagnosis	🟠 Emerging (ongoing validation)
**Local Field Potentials (LFPs)**	Intraoperative Electrophysiology	Advanced	Adaptive DBS programming	🟢 Clinical

Supplementary Table S2. Faculty contributions

Faculty	Expertise	Course lecture topic	Manuscript contribution
**Roberto Cilia**	Movement disorders; clinical translation; molecular imaging.	Course Direction; Chair	Overall coordination; drafting the first manuscript draft including cross-sectional integration
**Dario Arnaldi**	Prodromal synucleinopathies; sleep medicine; iRBD risk stratification	Prodromal stages of Synucleinopathies: Sleep and Glymphatic dysfunction	Section 4. *4.1. From Prodromal to Overt Clinical stages*
**Bénédicte Ballanger**	Noradrenergic system; PET/MRI biomarkers; non-motor symptoms	Noradrenergic Dysfunction: Molecular and MRI Biomarkers	Section 1 *1.2. Noradrenergic Dysfunction*
**Roberto Ceravolo**	Dopaminergic molecular imaging; SPECT/PET biomarkers	Dopaminergic Dysfunction: Molecular Biomarkers	Section 1 *1.1. Dopaminergic Dysfunction - Molecular Imaging*
**Rosa De Micco**	Network neuroscience; connectomics; progression modeling	Staging Parkinson's Disease: A Network Perspective	Section 4 *4.3. Network-driven conceptualization of PD*
**Angelo Del Sole**	Amyloid imaging; mixed pathologies; nuclear medicine	Imaging Amyloid and copathology; Chair (molecular imaging session)	Section 2 *2.3. Imaging Amyloid and Co-pathology*
**Roberto Eleopra**	Movement disorders; clinical phenotyping; advanced therapies	Chair (session discussion)	Editorial contribution; clinical integration across sections
**Hironobu Endo**	α-synuclein imaging; PET tracer development	Imaging Alpha-synuclein pathology	Section 2 *2.1. Imaging α-Synuclein Pathology*
**Alfonso Fasano**	Surgical therapies; DBS and lesioning; advanced PD management	Management of Parkinson's disease: surgical approaches	Section 4 *4.6. Management of PD: pharmacological and surgical approaches - Surgical approaches*
**Merle C. Hoenig**	Motor/cognitive reserve; resilience/compensation; neuroimaging correlates	The concept of Motor and Cognitive reserve vs. compensation in Neurodegenerative disorders	Section 4 *4.2. The Concept of Motor and Cognitive Resilience vs. Compensation in*

			Neurodegenerative Disorders: Two Sides of the Same Coin?
**Jacob Horsager**	Cholinergic dysfunction; PD subtypes; brain-first/body-first model	Cholinergic Dysfunction: Molecular Biomarkers	Section 1<br>*1.3. Cholinergic Dysfunction — Molecular Imaging*<br>Section 3<br>*3.5. Brain-First vs. Body-First: A Model to Reconcile Clinical, Imaging, and Pathological Heterogeneity.*
**Stéphane Lehéricy**	MRI biomarkers; neuromelanin/iron-sensitive MRI; nigrostriatal imaging	Dopaminergic Dysfunction: MRI Biomarkers	Section 1<br>*1.1. Dopaminergic Dysfunction - Magnetic Resonance Imaging*
**Valentina Leta**	Pharmacological therapy; infusion strategies; levodopa pharmacokinetics	Management of Parkinson's disease: oral and infusion pharmacological approaches	Section 4<br>*4.6. Management of PD: pharmacological and surgical approaches - Oral and infusion pharmacological approaches*
**Fabio Moda**	Seed amplification assays; translational biomarkers	Alpha-Synuclein Seed Amplification Assay: Present and Future	Section 3<br>*3.2. aSyn Seed Amplification Assays: Present and Future*
**Maria Nolano**	Skin biopsy; peripheral neuropathology; autonomic involvement	Update on the role Skin Biopsy in the diagnosis of Parkinsonism	Section 3<br>*3.3. The Role Skin Biopsy in the Diagnosis of Parkinsonism*
**Tiago F. Outeiro**	α-synuclein biology; therapeutic pipelines; disease mechanisms	Alpha-Synuclein: New discoveries and potential therapeutic applications	Section 3<br>*3.1. aSyn Biology: From Aggregates to Therapeutic Strategies*
**Laura Parkkinen**	Neuropathology; co-pathologies in PD and parkinsonism	The Role of Copathology in Parkinson's disease: the Neuropathologist's Perspective; Chair (pathology-focused session)	Section 3<br>*3.4. The Role of Co-pathologies in PD: The Neuropathologist's Perspective*
**Nicola Pavese**	Serotonergic imaging; neuropsychiatric and motor complications	Serotonergic Dysfunction: Molecular Biomarkers	Section 1<br>*1.4. Serotonergic Dysfunction*

**Andrea Quattrone**	MRI in atypical parkinsonism; differential diagnosis; trial endpoints	Imaging Biomarkers in Atypical Parkinsonian Disorders	Section 4<br>*4.5. MRI Biomarkers in Atypical Parkinsonian Disorders*
**Nicola J. Ray**	Cholinergic networks; MRI biomarkers; cognition/gait correlates	Cholinergic Dysfunction: MRI Biomarkers	Section 1<br>*1.3. Cholinergic Dysfunction — Magnetic Resonance Imaging*
**Martin M. Reich**	DBS connectomics; neuroimaging-guided targeting/programming	Updates on Neuroimaging approaches in Deep Brain Stimulation	Section 4<br>*4.6. Management of PD: pharmacological and surgical approaches - Neuroimaging approaches in DBS*
**Irena Rektorova**	Cognitive impairment imaging; MRI markers in Lewy body diseases	Imaging Cognitive Impairment; Chair (molecular imaging session)	Section 4<br>*4.4. MRI for tracking Cognitive Impairment in Lewy Body Diseases*
**Antonio P. Strafella**	SPECT & PET molecular imaging; TSPO PET;	Imaging Neuroinflammation; Chair (molecular imaging session)	Section 2<br>*2.4. Imaging Neuroinflammation*
**Fabrizio Tagliavini**	Neuropathology; neurodegenerative disease mechanisms	Chair (pathology-focused session)	Editorial contribution; pathology integration across sections
**Alessandro Tessitore**	Functional connectivity; PD networks; imaging signatures	Chair (imaging sessions)	Editorial contribution; MRI imaging and functional connectivity
**Thilo van Eimeren**	Tau imaging; multimodal neuroimaging; course direction	Imaging Tau pathology Course Direction; Chair	Section 2<br>*2.2. Imaging Tau Pathology*

Abbreviations: DBS, deep brain stimulation; iRBD, isolated/idiopathic REM sleep behavior disorder; MRI, magnetic resonance imaging; PET, positron emission tomography; SPECT, single photon emission computed tomography; TSPO, 18-kDa translocator protein.
